# Potential anti-neuroinflammatory NF-кB inhibitors based on 3,4-dihydronaphthalen-1(2*H*)-one derivatives

**DOI:** 10.1080/14756366.2020.1804899

**Published:** 2020-08-11

**Authors:** Yue Sun, Yan-Qiu Zhou, Yin-Kai Liu, Hong-Qin Zhang, Gui-Ge Hou, Qing-Guo Meng, Yun Hou

**Affiliations:** a The Key Laboratory of Prescription Effect and Clinical Evaluation of State Administration of Traditional Chinese Medicine of China, School of Pharmacy, Binzhou Medical University, Yantai, P. R. China; b School of Basic Medical Sciences, Binzhou Medical University, Yantai, P. R. China; c School of Pharmacy, Yantai University, Yantai, P. R. China

**Keywords:** 3,4-Dihydronaphthalen-1(2H)-one, anti-neuroinflammation, microglia polarisation, NF-кB inhibitor

## Abstract

Nuclear factor kappa B (NF-*к*B) inhibition represents a new therapeutic strategy for the treatment of neuroinflammatory diseases. In this study, a series of 3,4-dihydronaphthalen-1(*2H*)-one (DHN; **6a-n, 7a-c**) derivatives were synthesised and characterised by NMR and HRMS. We assessed the toxicity and anti-neuroinflammatory properties of these compounds and found that **6m** showed the greatest anti-neuroinflammatory properties, with relatively low toxicity. Specifically, **6m** significantly reduced reactive oxygen species production, down-regulated the expression of NOD-like receptor pyrin domain-containing protein 3 (NLRP3), apoptosis-associated speck-like protein containing a CARD (ASC), and caspase-1 and prevented lipopolysaccharide-stimulated BV2 microglia cells polarisation towards an M1 phenotype. Furthermore, **6m** significantly decreased I*κ*B*α* and NF-*к*B p65 phosphorylation, thus inhibiting the NF-*к*B signalling pathway. This suggests that **6m** may be explored as a functional anti-neuroinflammatory agent for the treatment of inflammatory diseases in the central nervous system, such as multiple sclerosis, traumatic brain injury, stroke and spinal cord injury.

## Introduction

Activated microglia-mediated inflammatory responses play an important role in the pathological development of inflammatory neurodegenerative diseases in the central nervous system (CNS)^1^^,^^2^. During inflammatory neurodegenerative diseases in CNS, the resident microglia become activated and polarised to a pro-inflammatory M1 phenotype^3^. It has been reported that pro-inflammatory cytokines (tumor necrosis factor (TNF-*α*), interleukin (IL)-6, IL-1*β*) secreted from M1 microglia increase blood-brain barrier (BBB) permeability by activating the nuclear factor kappa B (NF-*к*B) signalling pathway^4–8^. Concomitantly, the disrupted BBB promotes glial activation to exacerbate CNS inflammation^9^. In addition, activated microglia can produce reactive oxygen species (ROS), which may indirectly induce neuroinflammation by activating NF-*κ*B^10^. Moreover, the production of ROS can activate the NOD-like receptor pyrin domain-containing protein 3 (NLRP3) inflammasome formation through NF-κB signalling pathway^11–13^, which induces the polarisation of M1 microglia and the secretion of pro-inflammatory cytokines to exacerbate neuroinflammation of CNS^14–17^. Therefore, developing an NF-*к*B inhibitor with anti-neuroinflammatory properties and low toxicity is a potential therapeutic strategy for treating inflammatory neurodegenerative CNS diseases^18^.

3,4-Dihydronaphthalen-1(2*H*)-one (DHN) derivatives with antitumor and anti-inflammatory activities have been investigated as novel modulators of allergic and inflammatory responses^19^^,^^20^ and as potential inhibitors of retinoic acid (RA)-metabolizing enzymes for the treatment of skin conditions and cancer. Recently, several benzylidene-substituted DHN derivatives were reported as potential Bcl-2 inhibitors^21^. Additionally, 4-amino derivatives of DHN were developed as anti-inflammatory agents that stabilise mast cells^22^. Furthermore, dihydronaphthalene-1,4-dione derivatives inhibit the acetyltransferases ATase1 and ATase2 in neurons and glial cells, and therefore may serve as novel agents to prevent Alzheimer’s disease and dementia^23^. However, DHN derivatives have rarely been developed as anti-neuroinflammatory drugs. In this study, a series of new benzylidene-substituted DHN derivatives were designed and synthesised through Claisen-Schmidt condensation reactions, then evaluated for the anti-neuroinflammatory activities.

## Experimental

### Materials and methods

Several aromatic aldehyde, 3-fluorobenzylbromide, 4-fluorobenzylbromide, 4-trifluoromethylbenzylbromide were purchased from Sinopharm Chemical Reagent Co. Ltd (Shanghai, China). They were used as obtained without further purification. (*E*)-2–(2-fluorobenzylidene)-7-methoxy-3,4-dihydronaphthalen-1(*2H*)-one (**6d**) and (*E*)-2–(4-fluorobenzylidene)-7-methoxy-3,4-dihydronaphthalen-1(*2H*)-one (**6f**) were obtained following a literature^24^. NMR data were collected using a Bruker Avance 400 MHz for ^1^H NMR with Chemical shifts *δ* relative to TMS, while ^13^C NMR data were collected at 100 MHz on a Bruker Avance 400 MHz spectrometer or 150 MHz on a Bruker Avance 600 MHz spectrometer. The HREIMS data were obtained on a Finnigan-MAT-95 mass spectrometer.

### Synthesis of 6a-n: general procedure

7-Methoxy-3,4-dihydronaphthalen-1(2*H*)-one (denoted as **5**) was obtained according to the literature^24^. **5** (0.53 g, 3.0 mmol) and aromatic aldehyde (3.0 mmol) were dissolved in 10 ml of methanol. After 3.0 ml 20% NaOH solution were added, the mixture was stirred for 3–5 h at ambient temperature (monitored by TLC). The solvents were then removed by pouring, and the residues were purified on a silica gel by column chromatography using petroleum ether/EtOAc (2:1, v/v) as the eluent to produce light yellow powders **6a-n**.

### Synthesis of 7a-c: general procedure

*p*-Hydroxybenzaldehyde (1.22 g, 0.1 mmol) and 3-fluorobenzylbromide (1.89 g, 0.1 mmol), 4-fluorobenzylbromide (1.89 g, 0.1 mmol), or 4-trifluoromethylbenzylbromide (2.39 g, 0.1 mmol) were dissolved in 50 ml of acetone. After anhydrous potassium carbonate (1.38 g, 0.3 mmol) was added, the mixtures were stirred at 60 °C for 2 h (monitored by TLC) and filtered to obtain filtrate. After removal of solvents under a vacuum, the residues were purified on a silica gel by column chromatography using petroleum ether/EtOAc (4:1, v/v) as the eluent to generate the intermediates: 4-((3-fluorobenzyl)oxy)benzaldehyde (**8a**), 4-((4-fluorobenzyl)oxy)benzaldehyde (**8 b**), and 4-((4-trifluoromethylbenzyl)oxy)benzaldehyde (**8c**). Compounds **5** (0.35 g, 2.0 mmol) and **8a** (0.46 g, 2.0 mmol), **8 b** (0.46 g, 2.0 mmol) or **8c** (0.56 g, 2.0 mmol) were dissolved in 15 ml of methanol. After 6.0 ml of NaOH solution at 20% were added, the mixtures were stirred for 4–5 h at ambient temperature (monitored by TLC). The solvents were removed by pouring, and the residues were purified on a silica gel by column chromatography using petroleum ether/EtOAc (4:1, v/v) as the eluent to produce light yellow powders **7a-c**.

### Single-Crystal structure determination of 6m

The **6m** single crystals were prepared by slow evaporation of methanol solution under ambient conditions. Suitable single crystal measured at 100 K on a SuperNova diffractometer (Dual, Cu at zero, Mo K*α* radiation, *λ* = 0.71073 Å) using an AtlasS2 programmes. Crystal data of **6m**: C_20_H_17_F_3_O_3_, *M* = 362.33, Triclinic, space group *P-1*, colourless block, *a* = 8.2051(7) Å, *b* = 9.5552(8) Å*, c* = 11.1032(10) Å, *α =*  70.670(8)°, *β* = 83.365(7)°, *γ* = 79.760(7)°*, V* = 806.84(13) Å^3^, Z = 2, *D*c = 1.491 g·cm^−3^, *µ*(Cu-Kα) = 0.122 mm^−1^, *T* = 100.00(10) K. 2492 unique reflections [*R*_int_ = 0.050]. Final *R*_1_ [with *I* > 2σ(I)] = 0.052, *wR*_2_ (all data) = 0.144. CCDC 1983271 (**6m**) contains the supplementary crystallographic data for this paper. Copies of the data can be obtained free of charge on application to CCDC, 12 Union Road, Cambridge CB2 1EZ, UK (fax: (+44)1223–336-033; e-mail: deposit@ccdc.cam.ac.uk).

### Toxicity determination with cell counting kit-8 (CCK-8) assay

The toxicity of DHN derivatives (**6a-n, 7a-c**) was screened using CCK-8 kits (Sigma-Aldrich, MO, USA). The synthesised compounds were initially dissolved in dimethylsulphoxide (DMSO). Experimental concentrations of DMSO were always 0.1% (v/v), and concentrations of DHNs were 10 µM.

BV2 microglial cells (ATCC, Manassas, VA, USA) were cultured in 96-well plates then treated with DHN derivatives for 24 h. Next, 10 µL of the CCK-8 solution were added and incubated for 2 h at 37 °C, then absorbance at 450 nm was measured using a microplate reader. The survival rate (%) of BV2 microglia treated with compounds (10 µM) was then calculated. Results are the average of three replicates and shown in Table 1.

**Table 1.** Toxicity and anti-inflammatory activity of target compounds.

No.	The survival rate (%) of BV2 microglia cells treated with compounds (10 μM) by CCK-8	Inhibition rate (%) against TNF-α treated with compounds (10 μM) + LPS (1 μg/mL)
**6a**	95.4 ± 3.4	68.4 ± 2.5
**6b**	92.0 ± 2.1	57.5 ± 2.8
**6c**	96.7 ± 3.6	48.2 ± 5.6
**6d**	86.9 ± 2.2	51.4 ± 1.6
**6e**	90.3 ± 1.5	53.0 ± 2.8
**6f**	93.1 ± 4.5	69.9 ± 3.2
**6g**	90.3 ± 3.7	44.0 ± 3.7
**6h**	91.3 ± 3.6	67.2 ± 3.2
**6i**	90.6 ± 2.7	72.6 ± 5.1
**6j**	91.4 ± 2.5	46.0 ± 0.4
**6k**	89.5 ± 2.0	36.5 ± 2.4
**6l**	92.8 ± 5.5	32.0 ± 4.8
**6m**	95.5 ± 2.1	80.5 ± 1.9
**6n**	88.4 ± 2.1	65.8 ± 1.7
**7a**	95.8 ± 2.6	45.2 ± 2.1
**7b**	92.2 ± 4.3	59.5 ± 1.7
**7c**	89.5 ± 1.7	48.6 ± 3.5
**PDTC (30 μM)**	–	58.1 ± 1.6
**LPS (1 μg/mL)**	–	0

PDTC was used as a positive control.

### Screening of anti-neuroinflammatory activities of DHN derivatives by enzyme-linked immunosorbent assay (ELISA)

The anti-neuroinflammatory activities of DHN derivatives (**6a-n, 7a-c**) were screened by enzyme-linked immunosorbent assay (ELISA). Cultured BV2 microglia were pre-treated with or without lipopolysaccharide (LPS) (1.0 µg/mL) for 2 h, then were treated with pyrrolidinedithiocarbamate (PDTC) (30 µM) or DHN derivatives (**6a-n, 7a-c**) for another 24 h at 37 °C. The supernatants were then collected for testing the secreted levels of TNF-α using an ELISA kit (R&D Systems, Madison, WI, USA) according to the manufacturer’s instructions. Inhibition rate (%) was calculated as the average of three replicates, with PDTC used as a positive control, and expressed in Table 1.

### Assessing blood-brain barrier permeability to 6m

The ADMET descriptors protocol in Discovery Studio 2017R2 software was used to calculate the BBB penetration of **6m**^25^^,^^26^. The 3 D minimum energy structure of **6m** was generated, and the ADMET model was used to obtain ADMET_PSA_2D, ADMET_AlogP98 values. The ellipses define regions where well-absorbed compounds are expected to be found. The prediction level was estimated based on the ADMET model. The parameter of BBB function was scored on a scale of 0–3 (0: good penetration; 1: moderate; 2: poor; 3: very poor).

### Determination of ROS production

BV2 microglia were pre-treated with or without LPS (1.0 µg/mL) for 2 h, then treated with **6m** (0, 5, or 10 µM) for another 24 h. Following this, cells were collected to detect ROS production using the 2,7-dichlorodihydrofluorescein diacetate (DCFH-DA) ROS assay kit (Jiancheng, Nanjing, China) with fluorescence-activated cell sorting (FACS) (Becton-Dickinson, NJ, USA).

### Determination the expression of NLRP3-related proteins by Western blotting

BV2 microglia were pre-treated with or without LPS (1.0 µg/mL) for 2 h, then treated with **6m** (0, 5, 10 or µM) for another 24 h. After washing with PBS, the cells were collected and lysed for 10% SDS-PAGE. Membranes containing transferred proteins were probed with primary antibodies (anti-NLRP3, anti-apoptosis-associated speck-like protein containing a CARD (ASC), anti-cysteine protease caspase-1 (Caspase-1), and anti-*β*-actin) (Cell Signalling Technology, Beverly, MA, USA) at 4 °C overnight then visualised using an enhanced chemiluminescence (ECL) detection kit (Dalian Meilun Biotecnology Co., LTD, Dalian, China).

### Measurement of cytokines secreted from LPS-stimulated BV2 microglia

BV2 microglia cultured in 96-well plates were pre-treated with or without LPS (1.0 µg/mL) for 2 h, then were treated with **6m** (0, 5, or 10 µM) for another 24 h. The supernatants were then collected to measure the secretion of pro-inflammatory (IL-6/IL-18/IL-1*β*) and anti-inflammatory (IL-10) cytokines using Quantikine immunoassay ELISA kits (R&D) according to the manufacturer’s instructions.

### Determination of LPS-stimulated BV2 microglia polarisation

BV2 microglia incubated in 6-well plates were pre-treated with or without LPS (1.0 µg/mL) for 2 h, then treated with **6m** (0, 5, or 10 µM) for another 24 h. The cells were then double-stained with anti-mouse CD86-PE (BioLegend, San Diego, CA, USA) and anti-mouse nitric oxide synthase (iNOS)-FITC (BioLegend), and double-stained with anti-mouse arginase-1 (Arg-1)-APC (eBiosciences, San Diego, CA, USA) and anti-mouse CD206-PerCP-Cy5 (eBiosciences) at 4 °C for 30 min in the dark. Cells were then washed and resuspended in PBS to evaluate M1 or M2 marker expression by FACS (Becton-Dickinson).

### Expression of NF-кB signalling-related proteins evaluated by Western blotting

BV2 microglia incubated in 6-well plates were pre-treated with or without LPS (1.0 µg/mL) for 2 h, then treated with **6m** (0, 5, or 10 µM) for another 24 h. Cells were collected and processed for 10% SDS-PAGE. Membranes containing transferred proteins were probed with primary antibodies (anti-I*к*B*α*, anti-*p*-I*к*B*α*, anti-NF-*κ*B p65, and anti-*p*-NF-*κ*B p65) (Cell Signalling) at 4 °C overnight and visualised using an ECL detection kit (Meilun Biotecnology).

## Results and discussion

### Synthesis and structural characterisation of DHN derivatives

**Scheme 1:**
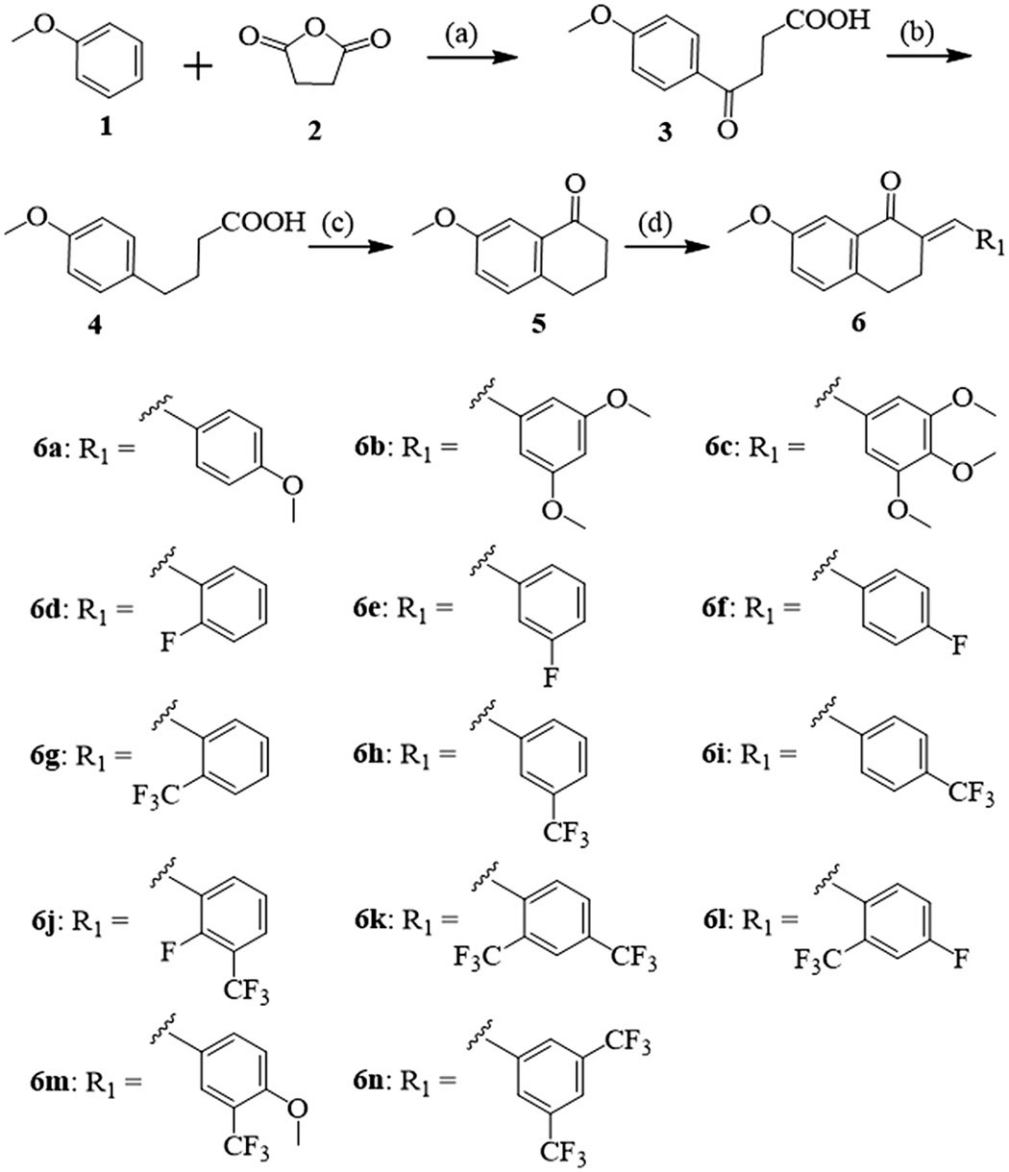
Synthetic route of compounds **6a-n**. Reagents and conditions: (a) AlCl_3_/CH_3_NO_2_, 0 °C, 3 h, 92%; (b) NH_2_NH_2_·H_2_O/KOH/DEG, 180 °C, 3 h, 86%; (c) PPA, 100 °C, 30 min, 65%; (d) Aromatic aldehyde, 20% NaOH (aq.), MeOH, 25 °C, 3–5 h.

**Scheme 2:**
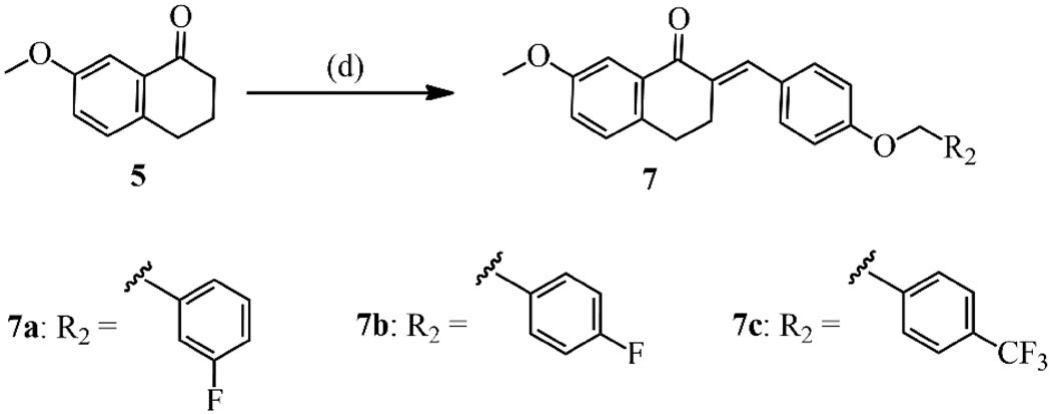
Synthetic route of compounds **7a**-**c**. Reagents and conditions: (a) aldehyde, 20% NaOH (aq.), MeOH, 25 °C, 3‒5 h.

In this study, the synthetic routes to 3,4-dihydronaphthalen-1(2*H*)-one derivatives are shown in Schemes 1 and 2. A key intermediate, 7-methoxy-3,4-dihydronaphthalen-1(2*H*)-one (**5**), was prepared with an overall yield of 48% using the three steps based on the method described in the literature^21^^,^^24^. First, anisole (**1**) and succinic anhydride (**2**) were combined to generate 4–(4-methoxyphenyl)-4-oxobutanoic acid (**3**) by Lewis acid catalysis (anhydrous AlCl_3_) with a 92% yield. Secondly, after Wolff-Kishner-Huang-Minlon reduction of **3**, 4-phenoxybutanoic acid **4** was generated with an 86% yield. Then, the key intermediate **5** was prepared for cyclisation in the presence of PPA with a lower yield of 65%. Lastly, **5** and several aromatic aldehydes were subjected to Claisen-Schmidt condensation to yield a series of new 3,4-dihydronaphthalen-1(2*H*)-one derivatives (**6a-n**). The yields of **6a-n** reached approximately 80–91%. During this last step, dry HCl, aqueous NaOH, or other bases can be chosen as the catalyst^27^. Considering its environmental friendliness and availability to laboratories, a 20% NaOH solution was selected as the catalyst (Scheme 1).

The structures of **6a-n** were characterised by NMR and MS (Supporting Information). Some selected spectral data are discussed below. In the ^1^H NMR spectra of our target compounds, the chemical shifts in the range of 7.89–7.65 ppm appear as a singlet attributed to the proton of *α,β*-unsaturated ketone pharmacophores. All compounds showed two groups of characteristic triplets from the two intra-annular methylene groups at *δ* 3.16–2.81 ppm. The singlets observed in the range of 5.21–5.09 ppm are likely the three protons of 7-methoxy in 3,4-dihydronaphthalen-1(2H)-one. In the ^13^C NMR spectra, the carbon atom of -C=O groups appeared in chemical shifts of approximately 187 ppm. Additionally, HRMS spectroscopy data further confirmed the accuracy of the structures of these compounds. In order to investigate the influence of the length of the substituent of 3,4-dihydronaphthalen-1(2*H*)-one derivatives on neuroinflammation, several benzyloxy-substituted arylaldehydes were selected to synthesise novel 3,4-dihydronaphthalen-1(2*H*)-one derivatives (**7a-c**). The yields of **7a-c** reached approximately 76–79%. All analyses showed the same results as described with **6a-n**, with the exception of additional singlets observed in the range of 5.21–5.09 ppm of the ^1^H NMR spectra of **7a-c**, corresponding to two methylene protons in the benzyloxy group. Corresponding NMR and HRMS data are shown in Supporting Information, which further confirm the accuracy of their structures.

**Figure 1:**
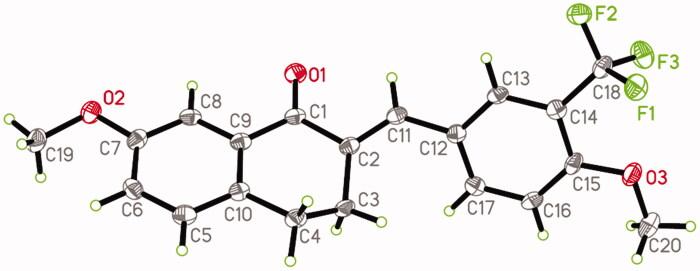
The ORTEP figure of **6 m** (displacement ellipsoids with 60% probability).

Single crystals of **6m** were prepared under ambient conditions, with crystallisation obtained via solvent evaporation in a methanol solution. Single-crystal structure analysis revealed that **6m** crystallised in the triclinic space group *P-1*. The ORTEP diagram is presented in Figure 1. There is only a drug molecule in the asymmetric unit. Compared to the C(2)=C(11) olefinic bonds, 4-methoxy-3-(trifluoromethyl)phenyl and carbonyl groups adopt the *E* stereochemistry^28^^,^^29^. Because of the distorting effect of 3,4-dihydrobenzo[*b*]oxepin-5(2*H*)-one, the 7-methoxyphenyl and 4-methoxy-3-(trifluoromethyl)phenyl groups are not coplanar with each other, with a dihedral angle of approximately 60.6(2)°. This twisted configuration may increase likelihood of interactions with bioactive molecules, for the purposes of creating more potent biological activity^30^.

### *In vitro* toxicity and anti-neuroinflammatory activity analysis

The toxicity against BV2 microglia for all synthesised compounds was tested by the CCK-8 assay. As shown in Table 1, the survival rates of BV2 microglia were above 85% for all synthesised compounds at a concentration of 10 µM. In particular, for **6a**, **6c**, **6m**, and **7a**, the survival rates of BV2 microglia can be greater than 95%. These results illustrate that all compounds have no obvious toxic effects on BV2 microglia *in vitro*. Furthermore, **6m** dose-dependently decreased the survival rate of BV2 microglia; however, **6m** (20 µM) decreased the survival rate to approximately 82% (Supporting Information Figure S1).

TNF-α is a commonly secreted pro-inflammatory cytokine from activated microglia^31^^,^^32^. To test the anti-inflammatory activities of our synthesised compounds, TNF-α secretion from LPS-pre-treated BV2 microglia with or without the synthesised compounds was detected by ELISA. PDTC (30 µM) was used as a positive control. As shown in Table 1, following PDTC treatment, TNF-α secretion was inhibited by approximately 58.1% from LPS-pre-treated BV2 microglia, compared to cells treated with LPS alone. When treated with 10 µM of the synthesised compounds, the TNF-α secretion inhibition rates showed variation between each compound. Intriguingly, among these compounds, **6a**, **6f**, **6 h**, **6i**, **6m**, **6n,** and **7 b** exhibited significantly higher anti-inflammatory activity than PDTC by inhibiting TNF-α secretion by 68.4%, 69.9%, 67.2%, 72.6%, 80.5%, 65.8%, and 59.5%, respectively.

When analysing the structure-activity relationship, 3,4-dihydronaphthalen-1(2H)-one group is the constant, primary active centre, while the different substitutions on the benzylidene should influence the variation in anti-inflammatory activity. For instance, **6a-c** have different numbers of electron-donating groups (-OMe), whereas trimethoxy substitutions result in a loss of anti-inflammatory activity. The anti-inflammatory activities followed the order: **6a** > **6b** > **6c**. Interestingly, a single *para*-substitution on the benzylidene was sufficient for this activity, such as **6a** (*para*-OMe), **6f** (*para*-F), and **6i** (*para*-CF_3_). Among the compounds with the same substitution (-F or -CF_3_), the anti-inflammatory activities followed the order *para*-substitution (**6f** and **6i**) > *meta*-substitution (**6e** and **6 h**) > *ortho*-substitution (**6d** and **6 g**), indicating that the position of substitutions affects the anti-inflammatory activity.

In order to examine the substituent effect of these synthesised compounds, double-substituted **6j**-**6n**, which had substitutions by strong electron-donating -OMe and electron-withdrawing -F and -CF_3_ groups, were synthesised then evaluated for anti-inflammatory activity. The results revealed that **6j**-**6l** with *ortho*-substitutions did not show remarkable anti-inflammatory activity. In contrast, *meta-* and *para*-substituted **6m** and double-*meta*-substituted **6n** displayed more significant anti-inflammatory activities, with **6m** specifically exhibiting the strongest anti-inflammatory activity, revealing that *meta-*CF_3_ and *para*-OMe substitutions could improve anti-inflammatory activity.

To investigate the influence of the length of the terminal substituent of 3,4-dihydronaphthalen-1(2*H*)-one derivatives, original arylaldehydes were changed to benzyloxy-substituted arylaldehydes, generating **7a-c**. However, these structural changes reduced the anti-inflammatory activity with inhibition rates ranging from 45–60% (Table 1).

In addition, fluorine and trifluoromethyl substitutions play an important role in bioactivity, as their lipophilicity can effectively increase the membrane permeability of drugs^33^. More importantly, the fluorine atom is a strong electronegative group, which can form multiple H-bonds with target proteins^34^. Additionally, stable C-F bonds can improve metabolic stability, and thus prolong the duration of action *in vivo*^35^. In this study, we focussed on fluorine and trifluoromethyl substituted DHNs.

Notably, we found that the most effective compound had relatively low toxicity (**6m**). ADMET properties were estimated using Discovery Studio 2017R2 to calculate the BBB penetration of **6m** in the CNS. The results showed the values of ADMET_PSA_2D and ADMET_AlogP98 were 35.16 and 5.172, respectively. The ellipses-defined regions of **6m** can be found in Supporting Information Figure S2. We confirmed that the ADMET_BBB_level value of **6m** was at level 0 (good penetrant). This indicates that compound **6m** can effectively penetrate the BBB in a suitable therapeutic concentration for further anti-neuroinflammatory studies. Based on the above evidence, **6m** (10 µM) was selected for a more-in-depth biological evaluation of anti-neuroinflammatory activity.

### 6m Decreases ROS production

**Figure 2.**
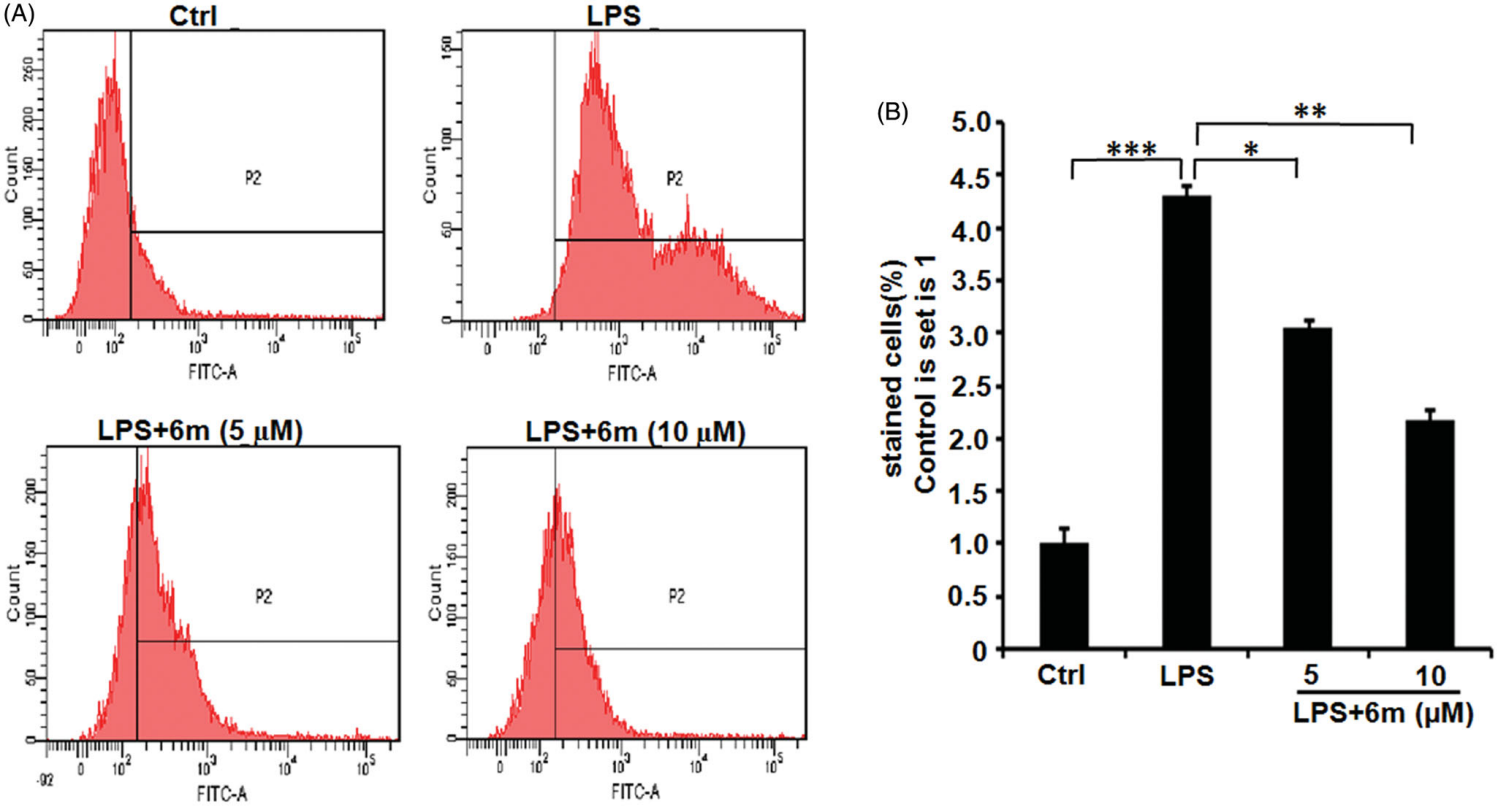
(A) The inhibitory effect of **6 m** on ROS production of LPS-stimulated BV2 microglia cells. (B) Statistical analysis of the number of DCF stained cells of LPS-stimulated BV2 microglia cells. Statistical significance is indicated: ∗*p* < .05, ∗∗*p* < .01, ∗∗∗*p* < .001 versus LPS group (one-way ANOVA followed by Dunnett’s test). The data are representative of three independent experiments.

ROS production due to oxidative stress was detected using the fluorescent probe 2,7-DCFH-DA^36^. During oxidative stress, 2,7-DCFH-DA can be oxidised by ROS to dichlorofluorescein (DCF), which can be detected fluorescently by FACS (Figure 2(A)). ROS production was quantified by counting the number of DCF-containing cells. As shown in Figure 2(B), LPS significantly induced ROS production in BV2 microglia, but **6m** dose-dependently and significantly inhibited this ROS production. These results suggest that **6m** can inhibit oxidative stress in LPS-stimulated BV2 microglia.

### 6m Reduces expression of inflammasome-related proteins

**Figure 3.**
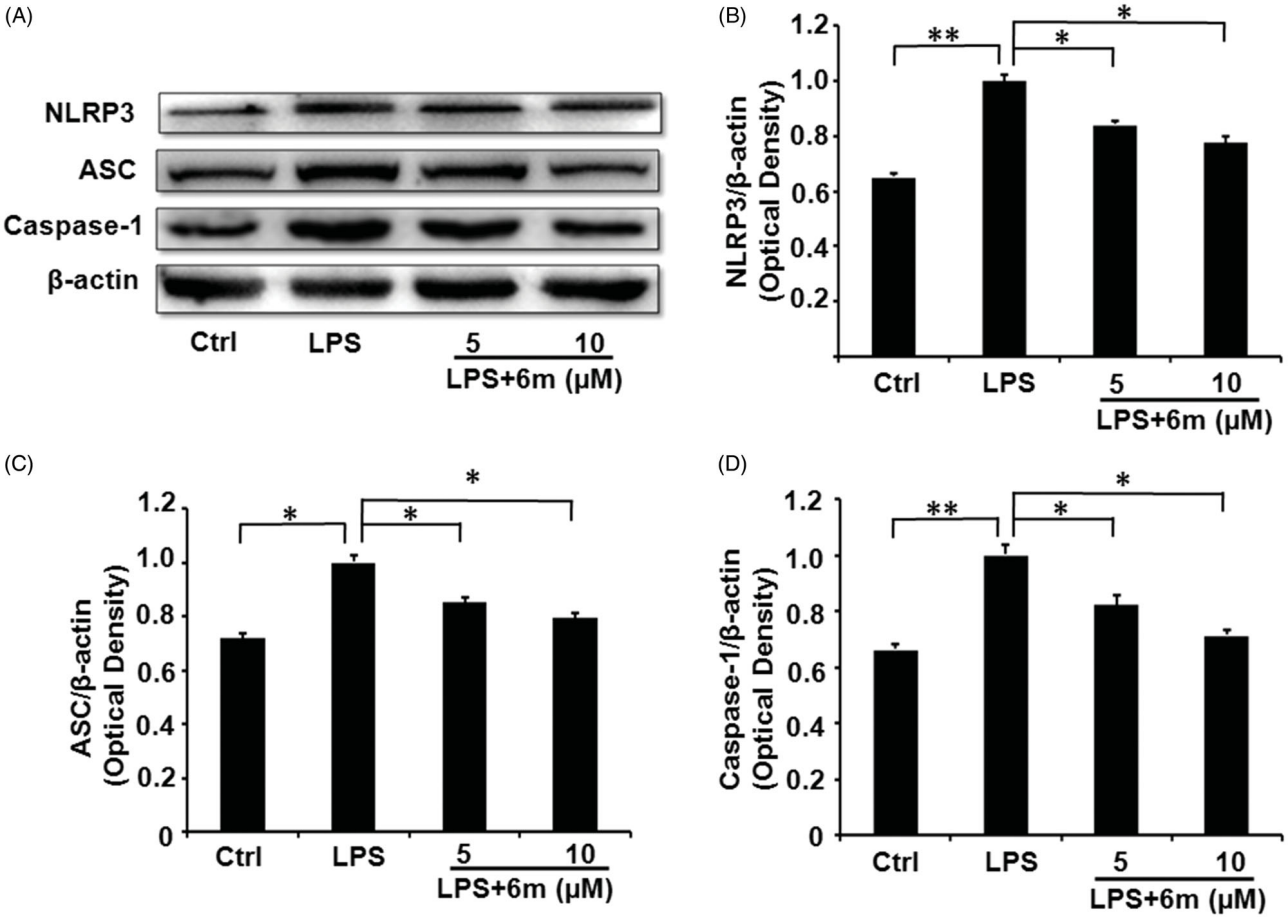
(A) The expression of NLRP3 inflammasome proteins reduced by **6 m**. (B) The expression of NLRP3 in LPS-stimulated BV2 microglia cells. (C) The expression of ASC in LPS-stimulated BV2 microglia cells. (D) The expression of Caspase-1 in LPS-stimulated BV2 microglia cells. Statistical significance is indicated: *p < .05, **p < .01 versus LPS group (one-way ANOVA followed by Dunnett’s test). The data are representative of three independent experiments.

The activation of NLRP3 leads to the formation of the NLRP3 inflammasome by combining with ASC and caspase-1^37^. This leads to the secretion of pro-inflammatory cytokines (such as TNF-*α*, IL-1*β*, IL-18, and IL-6) from activated microglia by activating the NF-κB signalling pathway, thus increasing neuroinflammation^16–19^, which contributes to neurological disease development and progression in the CNS^38^^,^^39^. Moreover, NLRP3 inflammasome inhibition shifts the M1 phenotype to an M2 phenotype by inhibiting the NF-κB signalling pathway^15^^,^^40^. As shown in Figure 3(A,B), LPS significantly increased the expression of NLRP3 in BV2 microglia, but this was dose-dependently and significantly reduced by **6m**. As a component of the NLRP3 inflammasome, ASC expression showed a similar expression pattern to NLRP3 in LPS-stimulated BV2 microglia cells with or without **6m** treatment (Figure 3(A,C)). Caspase-1, another component of the NLRP3 inflammasome, also showed a similar expression pattern to NLRP3 in LPS-stimulated BV2 microglia with or without **6m** treatment **(**Figure 3(A,D)). These results suggest that **6m** is a competitive inhibitor for NLRP3 inflammasome activation.

### 6m Modulates pro- and anti-inflammatory cytokine secretion

**Figure 4.**
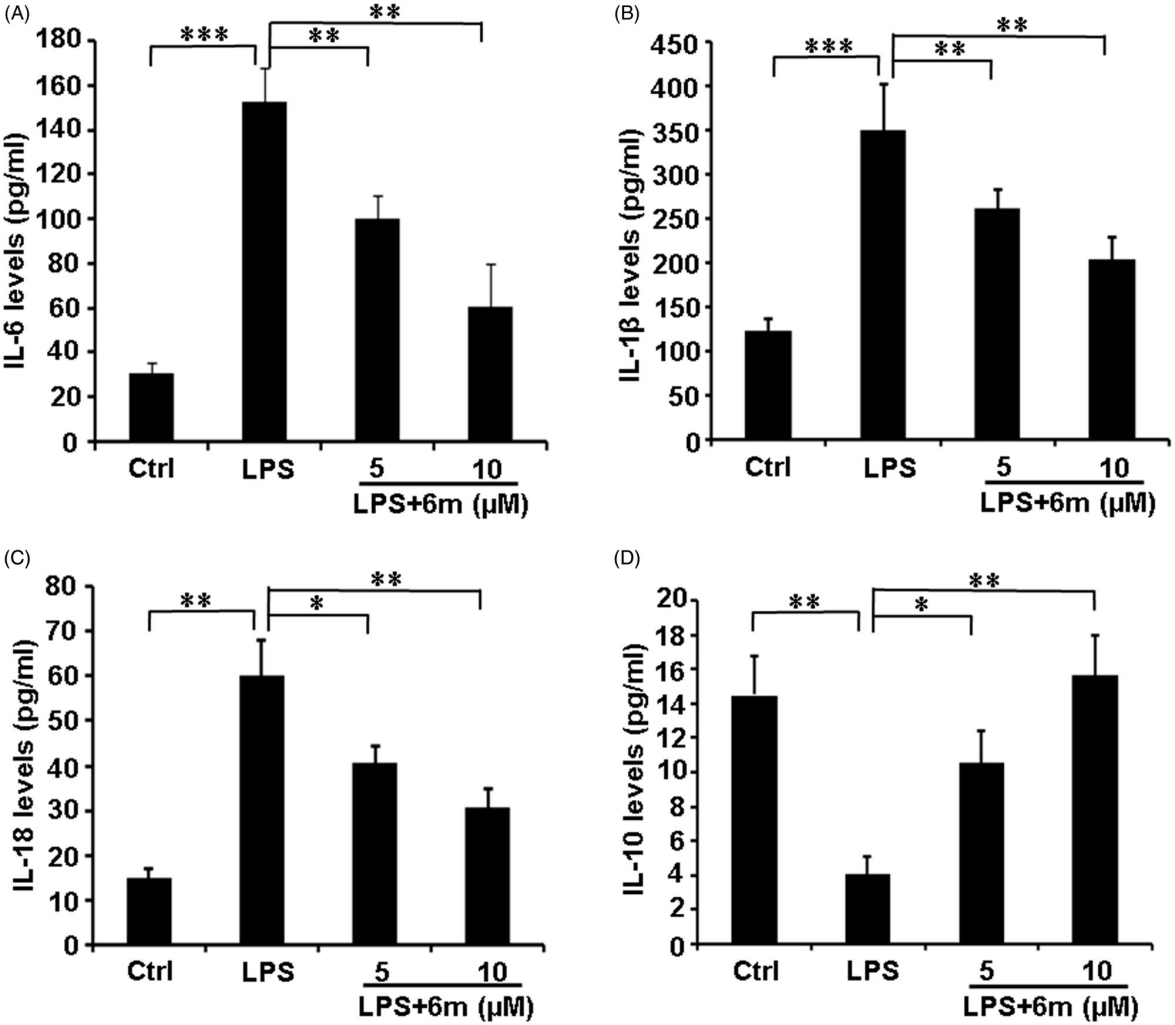
The effects of **6 m** on the secretion of cytokines from LPS-stimulated BV2 microglia cells. (A) Secretion of IL-6 from LPS-stimulated BV2 microglia cells. (B) Secretion of IL-1*β* from LPS-stimulated BV2 microglia cells. (C) Secretion of IL-18 from LPS-stimulated BV2 microglia cells. (D) Secretion of IL-10 from LPS-stimulated BV2 microglia cells. Statistical significance is indicated: *p < .05, **p < .01, ***p < .001 versus LPS group (one-way ANOVA followed by Dunnett’s test). The data are representative of three independent experiments.

M1 microglia play a critical role by secreting pro-inflammatory cytokines (TNF-α, IL-1β, IL-18, IL-6) to exacerbate neuroinflammation in the CNS^5^. Conversely, M2 microglia, the other of the two well-established microglia phenotypes, have an anti-inflammatory function by secreting IL-10^15^^,^^40^. As shown in Figure 4, LPS significantly increased the secretion of pro-inflammatory cytokines (IL-1β, IL-18, and IL-6), but decreased the secretion of the anti-inflammatory cytokine (IL-10) from BV2 microglia. However, **6m** significantly down-regulated the expression of these pro-inflammatory cytokines (IL-1β, IL-18, and IL-6), and increased the secretion of the anti-inflammatory cytokine (IL-10) from these cells. These results indicate that **6m** balances the secretion of pro- and anti-inflammatory cytokines to play an anti-inflammatory function.

### 6m Decreases LPS-stimulated M1 polarisation in BV2 microglia

**Figure 5.**
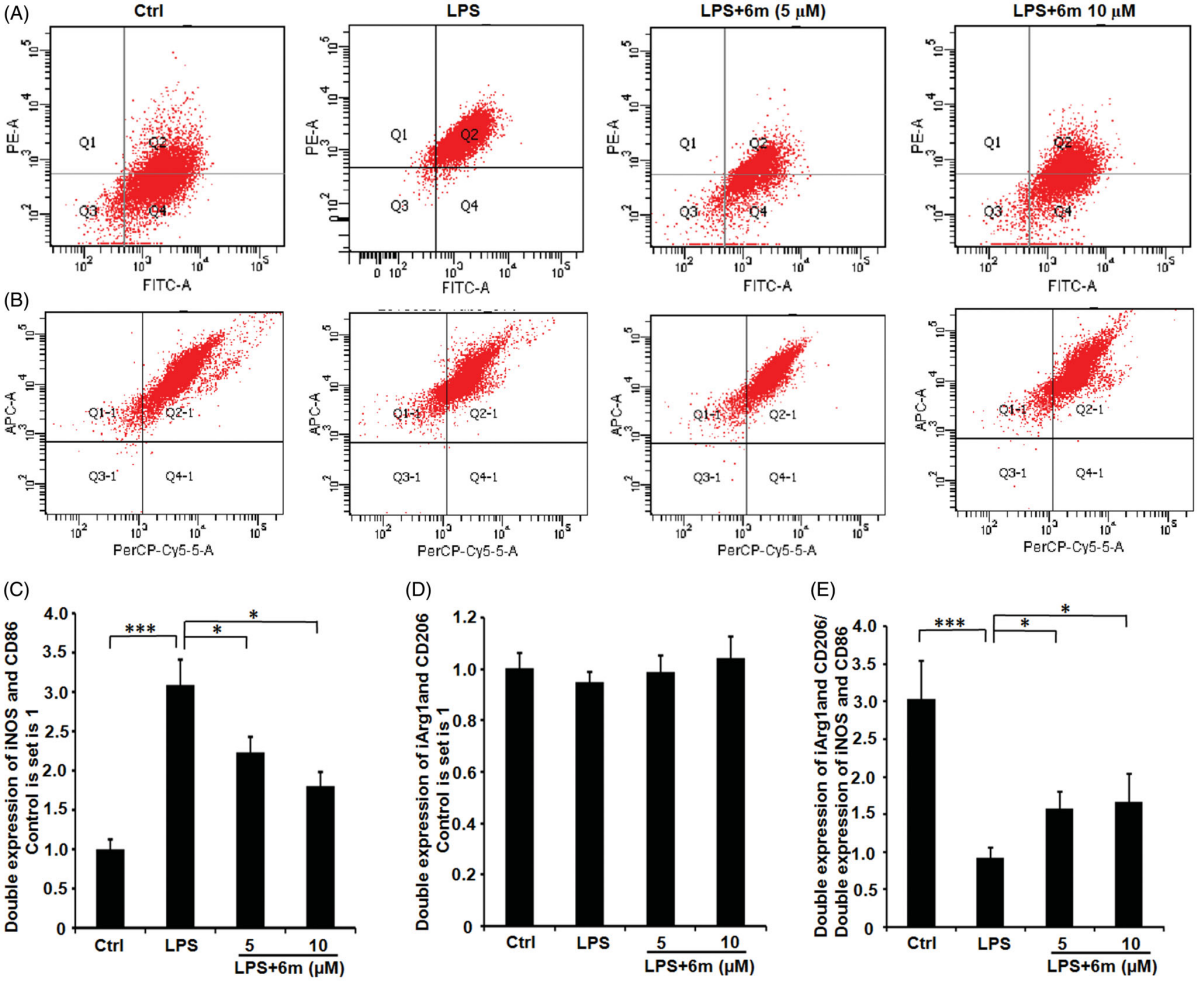
The effect of **6 m** on the polarisation of LPS-stimulated BV2 microglia cells by flow cytometry. (A) iNOS and CD86 expression in LPS-stimulated BV2 microglia cells. (B) CD206 and Arg 1 expression in LPS-stimulated BV2 microglia cells. The number of (C) double stained iNOS and CD86, (D) double stained CD206 and Arg 1, and (E) relative expression of double stained (iNOS and CD86) and double stained (CD206 and Arg1) cells of LPS-stimulated BV2 microglia cells. Statistical significance is indicated: *p < .05, ***p < .01 versus LPS group (one-way ANOVA followed by Dunnett’s test). The data are representative of three independent experiments.

LPS induces M1 polarisation, which can be detected through the expression of M1 phenotype markers (iNOS, CD68)^14^. As shown in Figure 5(A,C), LPS significantly induced the BV2 microglia to polarise to an M1 phenotype indicated by the numbers of iNOS- and CD86-expressing BV2 microglia. However, **6m** significantly inhibited this LPS-induced iNOS and CD86 expression. Even though **6m** did not significantly up-regulate the number of CD206- and Arg-1-expressing cells, the ratio of double-labeled CD206- and Arg-1-cells to double-labeled iNOS- and CD86-expressing cells is significantly increased by **6m** in LPS-stimulated BV2 microglia (Figure 5(B,D,E)). Taken together, **6m** decreases M1 polarisation of LPS-stimulated BV2 microglia to alleviate neuroinflammation.

### 6m Inhibits NF-кB activation

**Figure 6.**
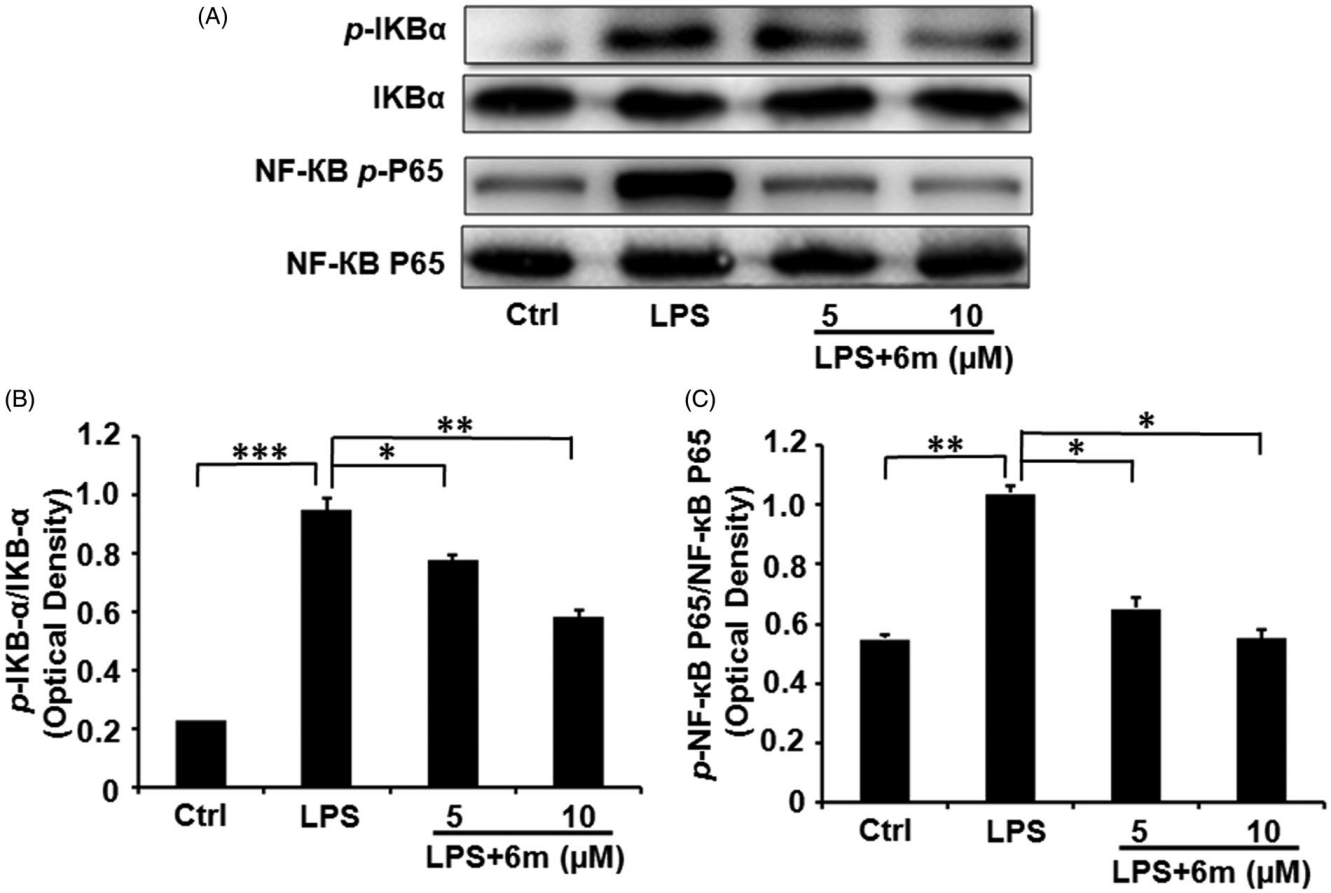
(A) The inhibitory effects of **6 m** on phosphorylation of I*κ*B*α* and p65 in LPS-stimulated BV2 microglia cells. (B) The relative expression of *p*-I*κ*B-*α* and I*κ*B-*α*. (C) The relative expression of *p*-NF-*κ*B and p65/NF-*κ*B p65. Statistical significance is indicated: *p < .05, **p < .01, ***p < .01 versus LPS group (one-way ANOVA followed by Dunnett’s test). The data are representative of three independent experiments.

To further confirm the effects of **6m** on inhibiting the NF-*к*B signalling pathway in LPS-stimulated BV2 microglia, we investigated the expression of I*κ*B*α, p-*I*κ*B*α*, NF-*κ*B p65 and *p*-NF-*κ*B p65 by Western blotting (Figure 6(A)). As shown in Figure 6(B,C,D), LPS significantly induced the phosphorylation of NF-*κ*B p65 and its upstream regulator (I*κ*B*α*) in BV2 microglia, in accordance with a previous study showing that LPS induces inflammatory cytokine secretion from BV2 microglia via NF-*κ*B signalling pathways^6^. However, **6m** significantly and dose-dependently decreased the levels of *p-*I*κ*B*α* and *p*-NF-*κ*B p65 in LPS-stimulated BV2 microglia. Collectively, these results reveal that **6m** significantly inhibits the NF-*к*B signalling pathway following LPS stimulation in BV2 microglia.

## Conclusions

In this study, a series of 3,4-dihydronaphthalen-1(2H)-one (DHN; **6a-n**, **7a-c**) derivatives were synthesised and characterised, then evaluated for their toxicity and anti-neuroinflammatory activity. The *meta-*CF_3_ and *para*-OMe substituted compound **6m** showed the greatest potential bioactivity with lower toxicity and the strongest anti-neuroinflammatory actions. Furthermore, **6m** significantly reduced ROS production, NLRP3 inflammasome expression, inflammatory cytokine secretion, and the number of M1 polarised cells following LPS stimulation in BV2 microglia. It also significantly inhibited the activation of the NF-*к*B signalling pathway. This study reveals that **6m** may be developed as a functional anti-neuroinflammatory agent for the treatment of inflammatory neurodegenerative disorders of the CNS, such as multiple sclerosis, Huntington’s disease, stroke, and traumatic brain injury.
